# Intra-individual impact of the COVID-19 pandemic on mental health and sleep in young adults

**DOI:** 10.1371/journal.pone.0276165

**Published:** 2022-10-27

**Authors:** Kelly J. Knickerbocker, Evelyn A. Cox, Luvna Dhawka, Kerri Woods, Krista K. Ingram

**Affiliations:** Department of Biology, Colgate University, Hamilton, NY, United States of America; The University of Manchester Division of Psychology and Mental Health, UNITED KINGDOM

## Abstract

The COVID-19 pandemic has posed unique academic, social, financial, and health-related challenges for young adults. While numerous studies have documented average increases in reported mental health issues in the general population, few have measured the magnitude of changes in mental health symptoms and sleep difficulties *within* individuals. Here, we measure the impact of the COVID-19 pandemic on mental health and sleep of university students pre- and mid-pandemic. Prior to the pandemic (Fall 2019), individuals (n = 23) were recruited to participate in an eight-day, comprehensive sleep study using Fitbit® actigraphy. Participants also completed detailed mental health and sleep surveys, including depression (BDI-II), anxiety (STAI), and sleep disturbance (PROMIS) surveys. One year later, these individuals repeated the study during the pandemic (Fall 2020); participants completed the original surveys and sleep study, in addition to a targeted survey on mental and sleep health due to the pandemic. Self-reported levels of anxiety, depression, and sleep disturbance, and sleep parameters, measured by actigraphy, were compared within the same individuals pre- and mid-pandemic. Self-report survey data revealed that three-quarters of participants experienced an increase in stress and anxiety due to the pandemic. In addition, intra-individual depression and anxiety symptoms increased to clinically significant levels within individuals from pre- to mid-pandemic. Over two-thirds of participants reported sleeping less, and more than half reported that their sleep health had worsened during the pandemic. Changes in sleep disturbance were positively associated with changes in depression and anxiety, reinforcing the robust relationship between poor sleep quality and mental health. Furthermore, individuals who reported greater sleep disturbance during the pandemic experienced lower relative proportions of both REM and deep sleep. The impact of the COVID-19 pandemic on university students is multi-faceted—mental health, sleep quality, and the amount of restorative sleep are negatively affected by the pandemic environment. These compounded effects exacerbate the health consequences of the pandemic and highlight a need for increased attention to the prevention and treatment of mental health disorders, particularly in vulnerable populations of young adults.

## Introduction

As a result of the COVID-19 pandemic, there has been a significant increase in symptoms of psychological distress among the general population [1, 2]. Specifically, it has been suggested that the pandemic is associated with both an increased prevalence of anxiety symptoms in the general population [2–5] and an overall increase in generalized anxiety disorder [4–8]. An increased prevalence of many factors relating to the pandemic, including threat of the disease in one’s self and loved ones, fear of infection, disruption of normal life or routine, social isolation, job loss or financial hardship, and change in sleep patterns, have been shown to exacerbate anxiety symptoms [3, 4, 9, 10]. Some studies report a marked increase in COVID-19-related anxiety in women [3, 11, 12]. In addition to increases in anxiety symptoms, multiple studies have found that the prevalence of depressive symptoms has also increased due to the pandemic [1, 2, 5, 6, 8]. This trend has been observed in both the general population as well as in individuals infected with SARS-CoV-2 [13]. Depression is often comorbid with anxiety, and individuals with anxiety symptoms have seen a higher prevalence of severe depressive symptoms during the pandemic [13]. Generally, among patients with preexisting psychiatric disorders (including depression and anxiety), the pandemic has been associated with the worsening of symptoms [1].

One demographic that has been particularly affected by the COVID-19 pandemic, in terms of mental health, are young adults and university students. Given the social and academic pressure that these individuals encounter on a daily basis, many university students experience mental health challenges in non-pandemic years [14–16]. In addition to the presence of those preexisting stressors, the COVID-19 pandemic has led to considerable disruptions in the way university students learn, live, and engage in social interactions. In combination with pandemic-related lifestyle changes, recent research has shown that the pandemic has negatively impacted the mental health of young adult student populations [12, 17, 18].

Previous research suggests that increased anxiety and depressive symptoms are associated with poor sleep quality in young, healthy adults [19–22]. Sleep plays an important role in emotional regulation, thus it is likely an important factor in determining mental health outcomes for at-risk populations such as university students. A recent study of Italian medical students demonstrated that individuals who judged that medical school influenced their ability to sleep properly were more likely to report depressive symptoms [23]. Worldwide, the COVID-19 pandemic has been suggested to increase the prevalence of poor sleep quality as well as the prevalence of sleep disorders [3, 5, 24–27]. In India, the pandemic was found to be associated with changes in sleep schedule, quantity, and quality of sleep in the general population [28]. In cross-sectional studies of university students, mental health and sleep quality are negatively affected during the pandemic; in one study, 71% of students reported increased anxiety and 86% reported increased sleep disturbance due to the pandemic [29]. As anxiety and depression have been significant correlates of sleep quality during the pandemic in the general population [3, 11, 24, 27], an analysis of these factors in the same individuals is essential to understanding the comprehensive effects of the pandemic on vulnerable populations. This is especially important given that quality of sleep has also been linked to immunity [30], and individuals with short sleep duration and poor sleep continuity are more likely to be susceptible to infectious diseases [31, 32].

The negative impacts on mental health clearly increase during the pandemic in the general population. However, it is difficult to understand the magnitude of these differences within individuals, or how negative affect intersects with sleep changes during the pandemic because previous studies focus on cross-sectional analyses and rarely use multiple measures of negative affect and/or sleep in the same study. In this study, we report the results of an intra-individual analysis of the impact of the COVID-19 pandemic on anxiety, depression, and sleep in young adults in the United States. Using a cohort of university students and the pandemic as a ‘natural intervention’, we compared self-reported depression, anxiety, and sleep parameters, as well as objective sleep data using a Fitbit® actigraphy, within individuals before and during the pandemic.

## Methods

### Participants

University students (n = 23 Caucasian individuals of European descent, 4 males, 19 females, median age: 19, range 18–21) who had previously participated in a sleep and mood study in the pre-pandemic fall semester of 2019 (October-November, 2019) were invited to take part in a continuation of the study during the following fall semester, 2020 (October-November, 2020), during the COVID-19 pandemic. The original study, designed to examine the effect of sleep patterns on mental health [33], included 78 individuals recruited as a convenience sample from an introductory biology course at Colgate University in Hamilton, New York, USA; the self-reported survey scores for depression, anxiety and sleep disturbance measures (see below for full description) did not differ between the subset of participants that retroactively agreed to complete the study again during the pandemic. No exclusion criteria were applied. In this study, we report only on intra-individual data measured in participants that completed the study both pre- and mid- pandemic, using the pandemic as a ‘natural intervention’ in a retrospective study. We recruited participants for the second part of the study with only one email request to avoid any coercion of students during the pandemic, resulting in a smaller than normal convenience sample for such studies. Participants included in the study reported socioeconomic status of wealthy (n = 8), upper middle class (n = 14), and lower middle class (n = 1). During the pandemic testing period, students arrived on campus to a two-week quarantine within their dorms with all classes online, meals delivered, and scheduled outdoor time for one hour. Following this quarantine period, students were COVID-19 tested biweekly, were required to wear masks at all times, and no social visitation was allowed between dormitory floors. Approximately half of courses were offered in person, with limited sporting or social activities prior to the study sample collection. The entire study took place at Colgate University. All methods were developed in agreement with the Declaration of Helsinki; procedures and consent forms were approved by the Institutional Review Board at Colgate University (ER-F19-06, ER-F20-11). Written informed consent was obtained from each participant prior to the study.

### Self-reported behavioral surveys

Participants completed computer-based surveys which included the Beck Depression Inventory (BDI-II) [34], State Trait Anxiety Inventory (STAI) [35], Patient-Reported Outcomes Measurement Information System (PROMIS) for Sleep Disturbance [36], reduced Morningness-Eveningness Questionnaire (MEQ) [37], and mid-sleep point on a free day (MSF), calculated using the Munich Chronotype Questionnaire (MCTQ; [38]. BDI-II is a psychometric test used to measure the presence and severity of clinical depression (one-week test–retest reliability of r = 0.93, an internal consistency α = 0.91 and high construct validity of 0.93 for college student samples), with scores ranging from 0 to 63. Participants are assessed as being minimally depressed (0–13), mildly depressed (14–19), moderately depressed (20–28) or severely depressed (> 28). The STAI measures both state and trait anxiety, with scores ranging from 20 to 80 (reliability of measure, r = 0.86). Participants with scores of 20–37 have no or low anxiety, 38–44 have moderate anxiety, and > 44 have high anxiety. The PROMIS is a self-assessed measurement of sleep disturbance (reliability exceeds r = 0.90), with scores ranging from 8 to 40. Participants with scores of 8 to 24 have no or slight sleep disturbance, 25–28 have mild sleep disturbance, 29–37 have moderate sleep disturbance, and ≥38 have severe sleep disturbance. Survey results were completed in Fall of 2019 and again in Fall of 2020 for each participant. Two additional questions were provided in the Fall 2020 surveys relating to sleep quality and mood in participants (see S1 File).

### Sleep data

Participants wore Fitbit® Versa 2 smartwatches to monitor personal activity for eight days in Fall of 2019 and again in Fall of 2020. The Fitbit measured seven sleep parameters: sleep duration (total minutes of sleep per night), percentage of deep sleep per night, percentage of light sleep per night, the percentage of REM sleep per night, onset and offset of sleep, number of awakenings in the night, and time spent in bed. Fitbit smartwatch devices use measures such as heart rate and movement to estimate sleep quality parameters and have been validated against comparable clinical sleep monitors, yielding similar results for sleep onset and offset, sleep duration and efficiency, and sleep structure [39, 40].

### Statistical analysis

Shapiro-Wilk and Kolmogorov-Smirnov tests were used to test the normality of distribution of behavioral survey scores and Fitbit sleep measures in our sample. Paired t-tests or Wilcoxon signed-rank tests were used to determine changes in our study variables between the two study periods. Bivariate Pearson or Spearman Rho correlations were used to detect relationships between our study variables, and multiple linear regression was used to analyze the relationship between the differences between pre- and mid-pandemic values of BDI-II (STAI as independent variable) and PROMIS as continuous outcome variables (with STAI scores, BDI scores and %REM and % deep sleep as independent variables for PROMIS); sex was tested as a covariate for all regressions. Odds ratios to test the likelihood of being depressed (BDI-II scores higher than 14) and anxious (STAI scores higher than 38) during mid-pandemic compared to pre-pandemic were calculated. One-way ANOVAs with post-hoc Tukey tests or Kruskal Wallis tests were used to test for differences in all study variables among anxious and depressed populations. Statistical analyses were conducted using SPSS using an alpha value of 0.05 to determine significance of analyses.

## Results

### Impact of pandemic on anxiety and depression

**Fig 1:**
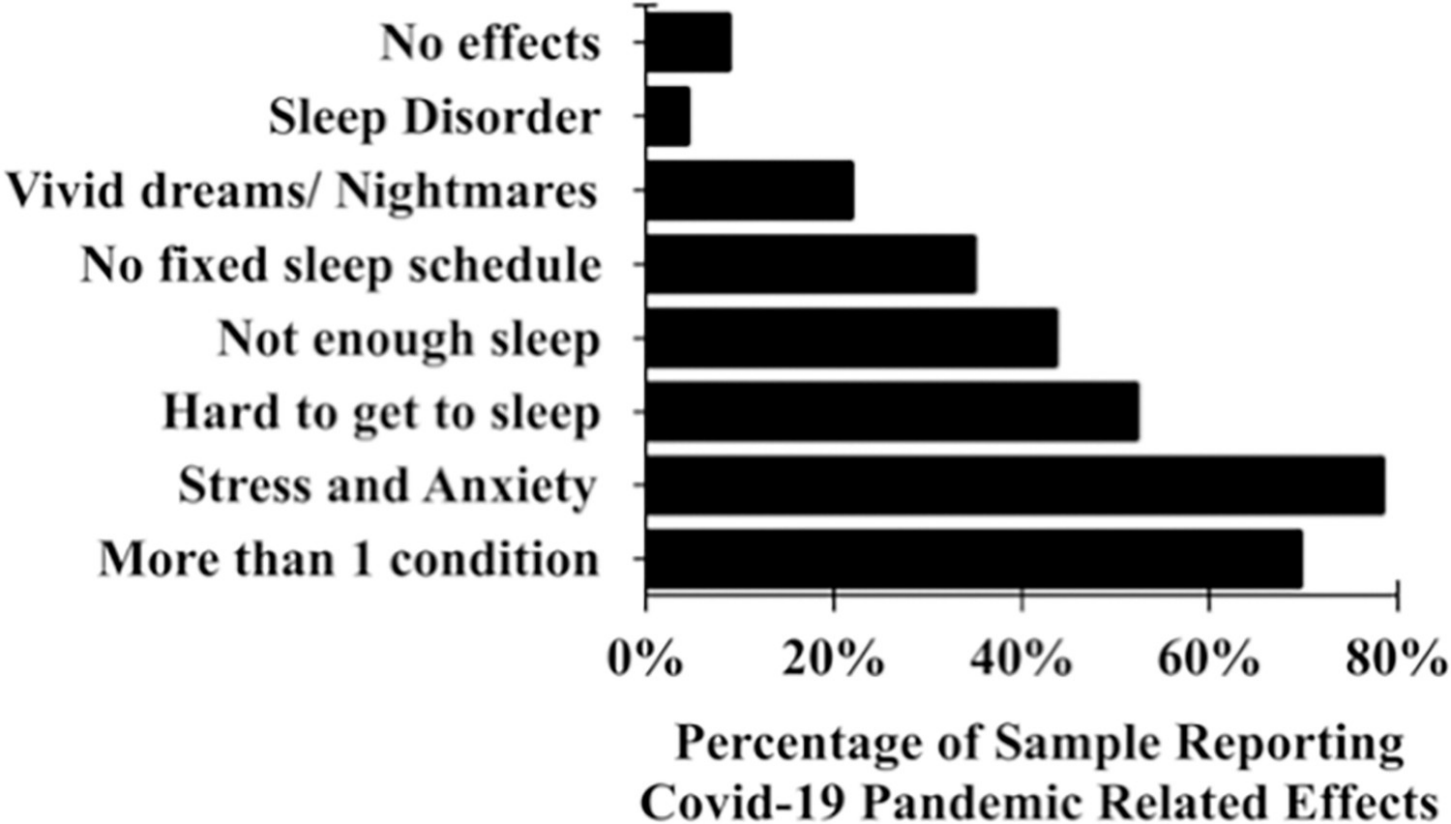
Proportion of participants reporting Covid-19 pandemic-related effects. 78% (18/23) of the participants reported that they felt an increase in stress and anxiety due to the pandemic; 52% (12/23) reported experiencing a harder time getting to sleep, 43% (10/23) felt that they were not getting enough sleep and 70% (16/21) reported suffering from more than one of the six effects listed, namely ‘sleep disorder’, ‘vivid dream/ nightmares’, ‘no fixed sleep schedule’, ‘not enough sleep’, ‘hard to get to sleep’ and ‘stress and anxiety’.

**Fig 2:**
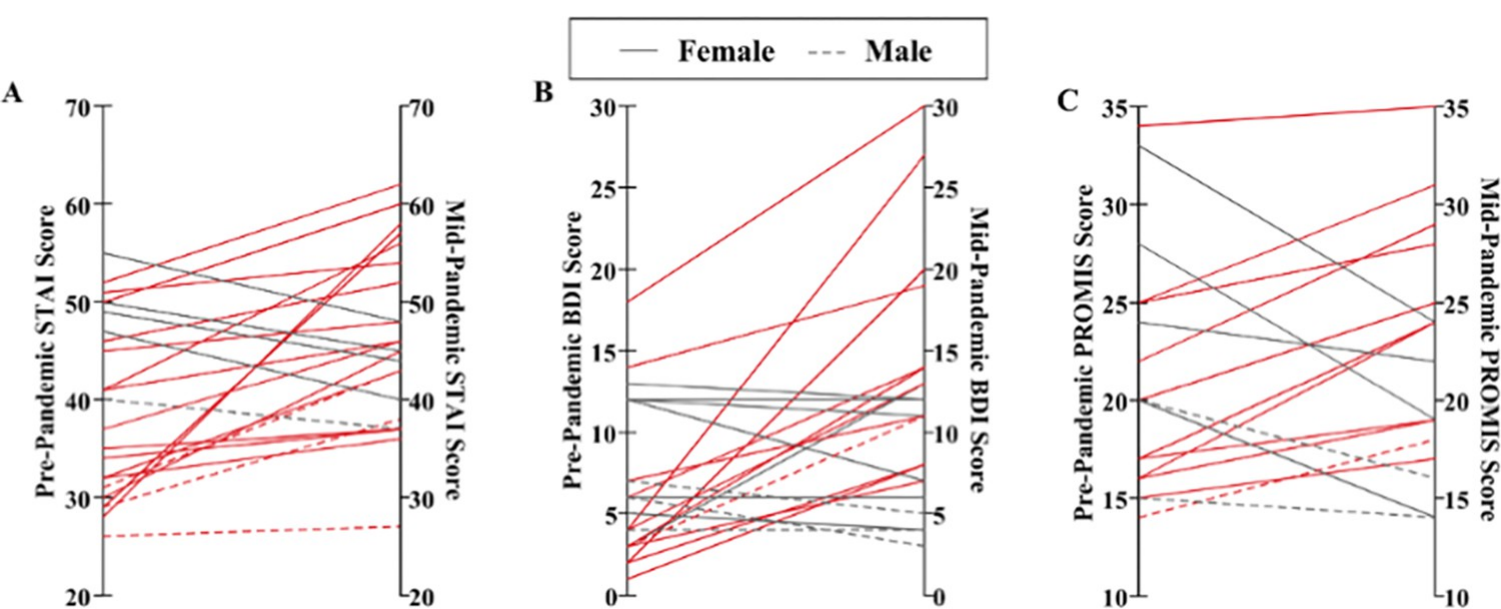
State Trait Anxiety Inventory (STAI) and BDI scores differ pre- and mid-pandemic. **A)**. STAI scores increased from pre- to mid-pandemic, (t (21) = -3.077, p = 0.006), with individuals being 4 times more likely to be anxious during mid-pandemic than during pre-pandemic (OR = 4.08, 95% CI: 1.11–15.02, Z = 2.12, p = 0.034). B) BDI scores increased from pre- to mid-pandemic (Z = -2.702, p = 0.007).

Seventy-eight percent (18/23) of participants reported that they felt increased stress and anxiety due to the Covid-19 pandemic (Fig 1). Intra-individual anxiety scores were significantly higher mid-pandemic than pre-pandemic (Fig 2; t (21) = -3.077, p = 0.006). Individuals were four times more likely to score as moderately anxious or above (STAI ≥ 38) during the pandemic relative to pre-pandemic (OR = 4.08, 95% CI: 1.11–15.02, Z = 2.12, p = 0.034).

Participants reported higher BDI scores during mid-pandemic relative to pre-pandemic (Z = -2.702, p = 0.007). The risk of reporting mild depression and above (BDI ≥ 14) was nearly four times higher during the pandemic but this value was not significant in this sample (OR = 3.71, 95% CI: 0.66–20.77, Z = 1.49, p = 0.136).

**Fig 3:**
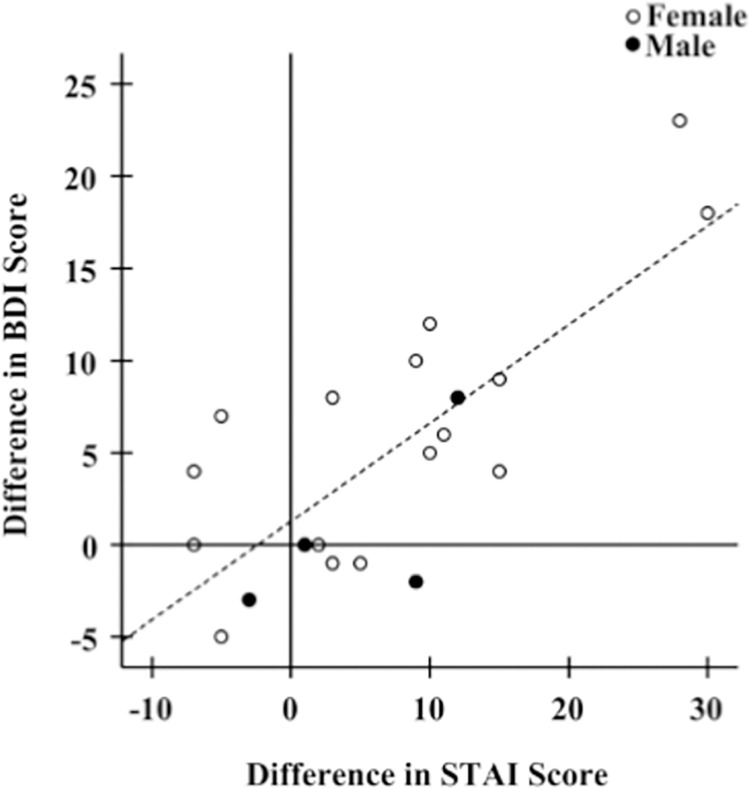
Association of differences in BDI scores between mid-pandemic and pre-pandemic with differences in STAI scores. Increasing relative anxiety scores (higher mid-pandemic scores relative to pre-pandemic scores) are associated with increases in depression scores (R^2^ = 0.632, β = 0.765, p < 0.001).

Adjusting for sex, the differences in depressive symptom scores between mid-pandemic and pre-pandemic were positively associated with differences in anxiety scores (Fig 3; Table 1, R^2^ = 0.632, p < 0.001).

**Table 1:** Regression analysis of differences between mid- and pre-pandemic depression symptoms (BDI) (as outcome) and anxiety (STAI).

Dependent/outcome: BDI (F’20 -F’19)							
Model	Variables entered	R^2^	B	SE (B)	β	t	p
**1**	STAI (F’20 -F’19)	0.585	0.535	0.103	0.765	5.178	**0.000**
**2**	STAI (F’20 -F’19)	0.632	0.521	0.100	0.745	5.189	**0.000**
	Gender		3.813	2.528	0.217	1.508	0.149

Model 1: unadjusted regression

Model 2: adjusted for sex

B: unstandardized coefficient

SE (B): standard error for coefficient B

β: standardized coefficient

### Impact of pandemic on self-reported sleep

Mid- pandemic, 68% (15/22) of our participants reported that they were sleeping less, 45% (10/22 reported that they were going to sleep later, and 59% (13/22 reported that their sleep health had gotten worse, relative to before the pandemic. Self-reported risk for sleep disturbance tended to be three times higher mid-pandemic relative to pre-pandemic, although this difference was not significant (OR = 3.04, 95% CI: 0.51–18.11, Z = 1.22, p = 0.223). Interestingly, PROMIS sleep disturbance scores reported during the pandemic did not differ from pre-pandemic scores (t (17) = -0.823, p = 0.422).

**Fig 4:**
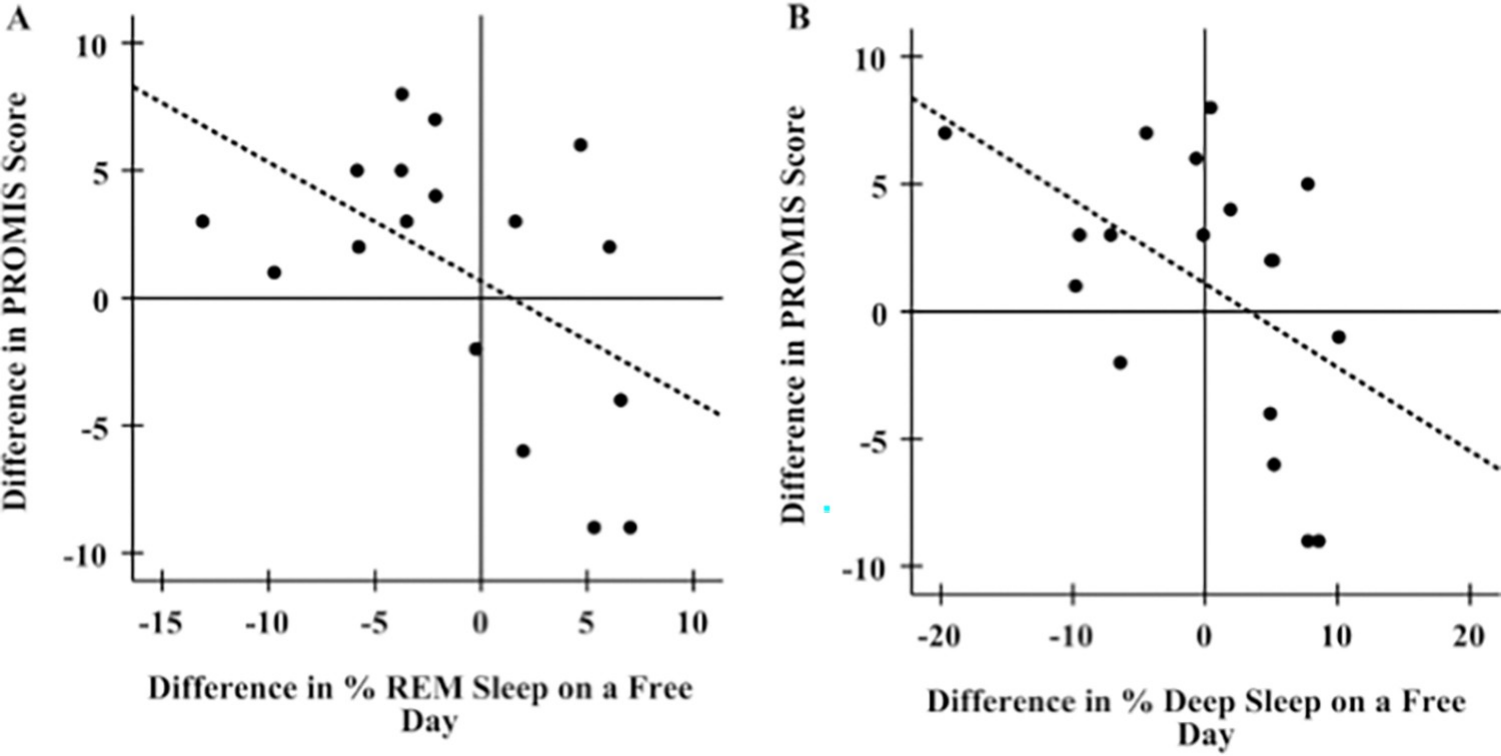
Association of the differences in PROMIS scores mid-pandemic versus pre-pandemic with differences in STAI and BDI scores. Increases in sleep disturbance scores during the pandemic are positively associated with increases in (A) anxiety scores (R^2^ = 0.258, β = 0.508, p = 0.045) and (B**)** BDI scores (R^2^ = 0.305, β = 0.552, p = 0.022).

**Fig 5:**
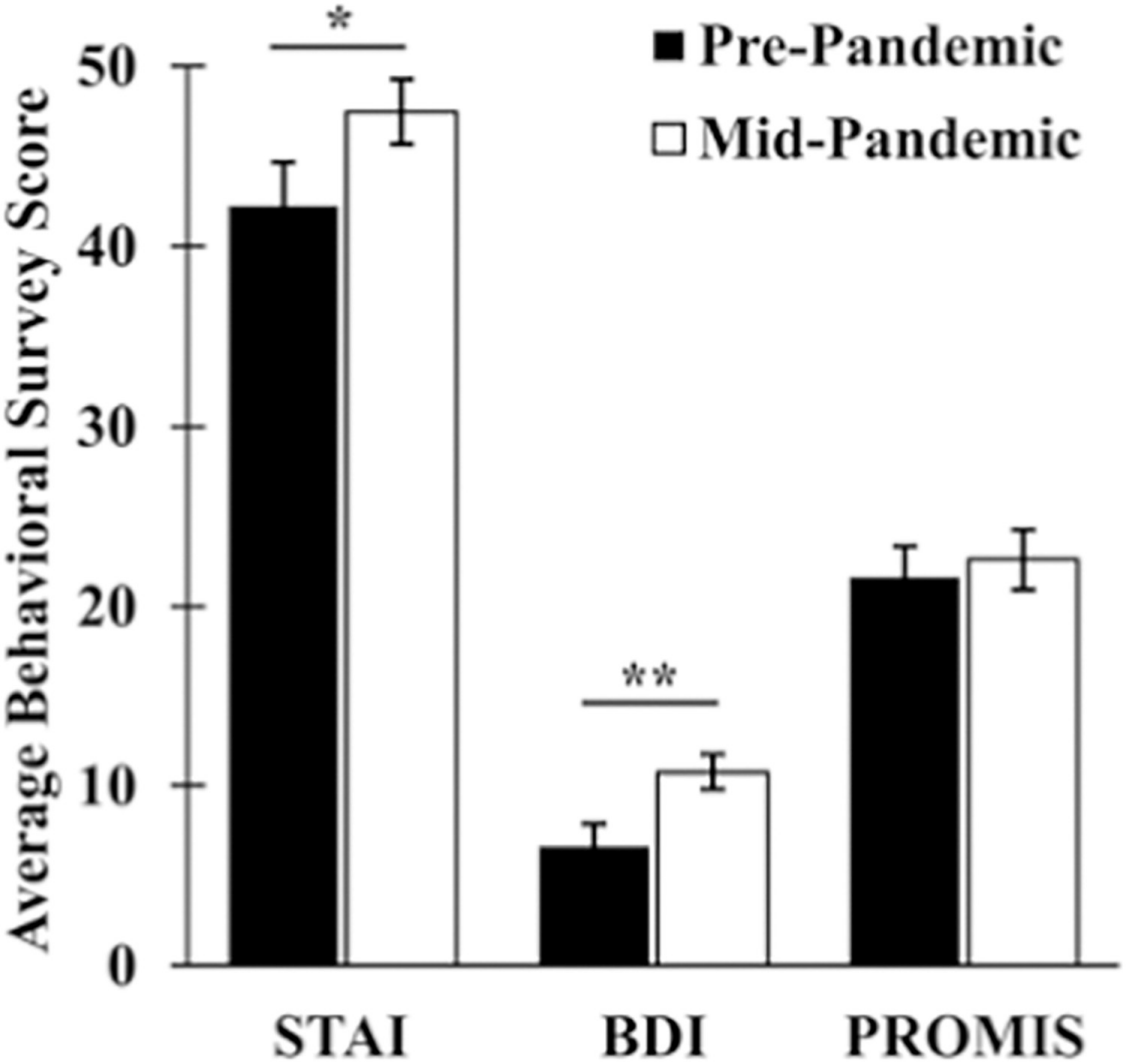
Average behavioral survey scores (STAI, BDI, PROMIS) in individuals reporting less sleep during pandemic. Individuals reporting that they spent less time sleeping during the pandemic compared to pre-pandemic reported significantly higher STAI (t(14) = -2.186, p = 0.046), and BDI (t(13) = -3.168, p = 0.007) scores during mid-pandemic relative to pre-pandemic. There was no significant change in PROMIS scores (t(12) = -0.800, p = 0.439).

Adjusting for sex, changes in sleep disturbance scores mid-pandemic relative to pre-pandemic measures may be weakly associated with changes in anxiety and depression scores (Fig 4; Table 2, STAI: R^2^ = 0.269, p = 0.050; BDI: R^2^ = 0.312, p = 0.033). In addition, individuals who reported that they got less sleep mid-pandemic relative to pre-pandemic also reported higher mid-pandemic depression and anxiety scores, but interestingly, not sleep disturbance scores (Fig 5; STAI: t (14) = -2.186, p = 0.046; BDI: t (13) = -3.168, p = 0.007; PROMIS: t (12) = -0.800, p = 0.439). No significant differences in mental health measures were observed for individuals reporting that they slept more mid-pandemic relative to pre-pandemic (STAI: t (3) = -2.587, p = 0.081; BDI: t (3) = -1.476, p = 0.236; PROMIS; t (1) = 0.059, p = 0.963).

**Table 2:** Regression analysis of differences between mid- and pre-pandemic sleep disturbance (PROMIS) (as outcome) and anxiety (STAI) and depression (BDI).

Dependent/outcome: PROMIS (F’20 -F’19)							
Model	Variables entered	R^2^	B	SE (B)	β	t	p
**1**	STAI (F’20 -F’19)	0.258	0.322	0.146	0.508	2.204	**0.045**
**2**	STAI (F’20 -F’19)	0.269	0.346	0.160	0.545	2.164	**0.050**
	Gender		-1.773	4.011	-0.111	-0.442	0.666
**1**	BDI (F’20 -F’19)	0.305	0.374	0.146	0.552	2.565	**0.022**
**2**	BDI (F’20 -F’19)	0.312	0.362	0.153	0.534	2.360	**0.033**
	Gender		1.049	2.679	0.089	0.392	0.701

Model 1: unadjusted regression

Model 2: adjusted for sex

B: unstandardized coefficient

SE (B): standard error for coefficient B

β: standardized coefficient

### Impact of pandemic on objective sleep measures

**Fig 6:**
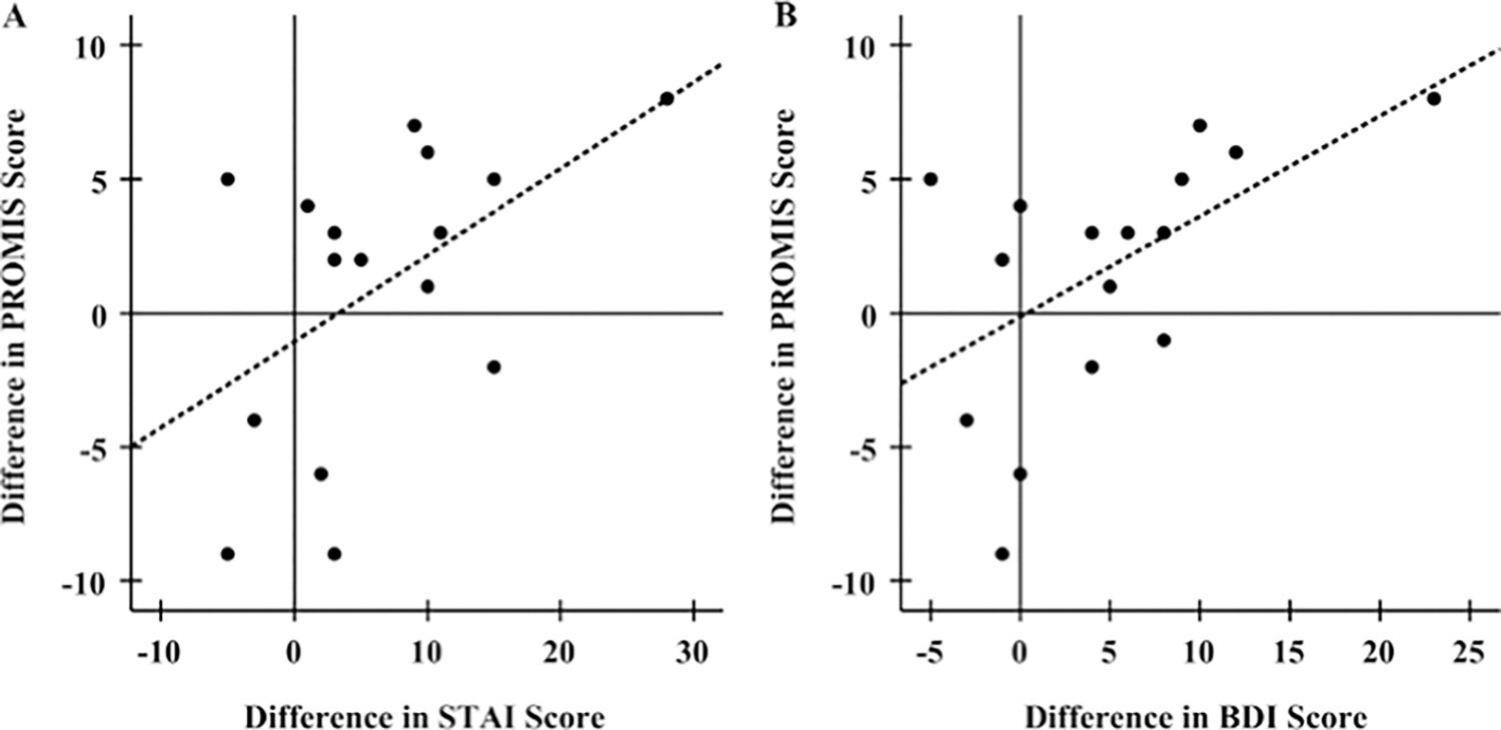
Association of mid-pandemic changes in PROMIS scores relative to pre-pandemic with changes in percentages of REM and deep sleep on free (non-work) days. A) Increasing PROMIS scores during pandemic are associated with decreases in the amount of REM sleep received during the pandemic relative to pre-pandemic (R^2^ = 0.269, β = -0.518, p = 0.033). B) Increasing PROMIS scores during pandemic are associated with decreases in the amount of deep sleep received during the pandemic relative to pre-pandemic (R^2^ = 0.248, β = -0.498, p = 0.035).

Overall, objective measures for sleep duration and the percentages of REM, deep and light sleep on free days via Fitbit analysis did not differ between mid-pandemic and pre-pandemic measures (Table 3, Sleep duration: Z = - 0.260, p = 0.795; % REM: Z = - 0.081, p = 0.935; % deep: Z = - 0.730, p = 0.465; % light: t (21) = 0.147, p = 0.885). However, an increase in sleep disturbance scores between mid- and pre-pandemic tended to be only weakly associated with a decrease in the percentage of REM and deep sleep (Fig 6; Table 4; %REM: R^2^ = 0.269, p = 0.041; % deep: R^2^ = 0.248, p = 0.047), suggesting that an increase in sleep disturbance mid-pandemic may be negatively correlated with a decrease in the amount of REM sleep and deep sleep.

**Table 3:** Average values for sleep parameters measured by Fitbit on free days (median (IQR)).

	Pre-Pandemic			Mid-Pandemic		
Fitbit Measure	Female (n = 18)	Male (n = 4)	Overall (n = 22)	Female (n = 18)	Male (n = 4)	Overall (n = 22)
Sleep duration	438.25 (98.40)	452.75 (104.90)	442.50 (97.60)	454.25 (67.0)	399.25 (118.10)	435.25 (64.80)
%REM sleep	19.66 (9.01)	16.09 (5.15)	18.80 (8.08)	19.21 (6.14)	20.33 (20.57)	19.75 (6.14)
%Deep sleep	16.10 (5.98)	16.23 (12.66)	16.10 (5.98)	18.60 (8.46)	17.86 (7.38)	17.86 (6.78)
%Light sleep	62.28 (16.51)	63.06 (18.32)	62.28 (15.00)	64.52 (17.98)	59.21 (10.14)	63.28 (16.19)

**Table 4:** Regression analysis of differences between mid- and pre-pandemic sleep disturbance (PROMIS) (as outcome) and Fitbit sleep measures (REM and deep sleep).

Dependent/outcome: PROMIS (F’20 -F’19)							
Model	Variables entered	R^2^	B	SE (B)	β	t	p
**1**	%REM (F’20 -F’19)	0.269	-0.466	0.199	-0.518	-2.348	**0.033**
**2**	%REM (F’20 -F’19)	0.269	-0.471	0.210	-0.524	-2.246	**0.041**
	Gender		-0.444	3.718	-0.028	-0.119	0.907
**1**	%deep (F’20 -F’19)	0.248	-0.328	0.143	-0.498	-2.300	**0.035**
**2**	%deep (F’20 -F’19)	0.250	-0.337	0.156	-0.512	-2.164	**0.047**
	Gender		-0.574	3.251	-0.042	-0.176	0.862

Model 1: unadjusted regression

Model 2: adjusted for sex

B: unstandardized coefficient

SE (B): standard error for coefficient B

β: standardized coefficient

## Discussion

Our results show marked intra-individual increases in anxiety and depressive symptoms following the natural intervention of the COVID-19 pandemic in university students, a population of young adults who are particularly vulnerable to mood disorders and sleep problems. Increases in COVID-related anxiety and self-reported sleep issues are correlated with higher levels of anxiety and depressive symptoms in the same individuals. Intra-individual increases in anxiety and depression are clinically relevant; more individuals report symptoms indicative of diagnostic levels of anxiety and major depressive disorder mid-pandemic relative to pre-pandemic. In addition, our results suggest that these inflated levels of negative affect may be associated with objective measures of sleep quality. Individuals reporting high levels of sleep disturbance during the pandemic experienced less deep sleep and REM sleep relative to overall sleep.

### Impact of pandemic on anxiety and depression

The COVID-19 pandemic has led to an increase in the prevalence of depressive and anxiety symptoms in the general population [1, 2] and particularly in young adults [12, 17, 18, 29]. University students typically experience high levels of academic, financial, and social stress, which makes them uniquely vulnerable to anxiety and depression [14–16], and such symptoms have been shown to be exacerbated during the COVID-19 pandemic [12, 17, 18]. Our results support these findings as nearly four-fifths of participants indicated that they have experienced increased levels of stress and anxiety due to the pandemic. Additionally, individuals are four times more likely to report clinical levels of anxiety during the pandemic. The increase in overall levels of anxiety may be associated with several factors such as financial burdens, health stressors, threat of the disease on self and loved ones, fear of infection, disruption of normal life and routine, social isolation, job loss, and change in sleep patterns [1, 3, 4, 9, 10].

Depressive symptoms also increased during the pandemic in our sample. Self-reported scores on the diagnostic BDI-II were significantly higher in the same individuals mid-pandemic relative to pre-pandemic. These results support recent studies on increased levels of depressive symptoms in cross-sectional studies of diverse populations [6, 8, 13]. As expected from previous reports detailing the comorbidity of depression and anxiety [41–43], we found a strong association between increases in depression and increases in anxiety within individuals. Although not statistically significant in this sample, our results suggest that individuals may be nearly four times more likely to report clinical levels of depressive symptoms during the pandemic.

### Sleep and mental health during Covid-19 pandemic

Using both self-report survey data and objective Fitbit™ sleep data, our results show interesting connections between sleep and mental health. According to survey data, 68% of the participants reported sleeping less, and 59% reported that their sleep health had gotten worse during the pandemic. Participants that reported worse sleep due to the pandemic also scored higher on the clinical anxiety and depression diagnostic surveys. Similarly, the significant association between the diagnostic sleep disturbance scores and depression and anxiety scores pre to mid pandemic suggest that increases in sleep disturbance and negative affect during the pandemic are linked. Our results support previous findings that subjective sleep quality can affect both psychological and neurological emotional state, which, in turn, could affect the processing of emotions and emotional empathy [44].

Although pandemic-related sleep disturbance did not increase in all individuals, those individuals who suffered from the most acute increase in sleep problems also had reported large increases in anxiety and depression. In addition, we found significant decreases in percent REM and deep sleep in individuals reporting high sleep disturbance scores during the pandemic relative to pre-pandemic. Notably, the observed decrease in percent REM and deep sleep as well as heightened sleep disturbances may have implications for the ability of individuals to mount a sufficient immune response. Given that sleep duration and quality is directly related to immune strength, the observed decrease in deep sleep among undergraduate students may have clinical implications beyond the scope of our study [30–32].

### Limitations

This study had several limitations, including the limited number of males in the sample which precluded inferences on sex differences in the study. In general, the small sample size does not allow us to fully investigate differences between sub-groups within the population (eg. sex, socioeconomic background, etc.) from which the sample is drawn. Because our participants were university students and primarily Caucasian of European descent, it may be difficult to generalize the results of the study to other populations, including those representing diverse backgrounds. Participants were originally recruited from an introductory Biology course as a convenience sample, and the inclusion of primarily STEM-interested students may have introduced a bias in the dataset. In addition, some of the significance values of individual statistical analyses would not retain significance if we corrected for multiple comparisons, and the small sample size limits robust interpretation of our multiple regression analyses. Although Fitbit sleep measures are robust relative to other actigraphy measures, the accuracy of sleep stage measurement is limited relative to polysomnography techniques. While we can speculate causality due to the retrospective nature of this study and the pandemic as a ‘natural intervention’, it is possible that changes in anxiety, depression, and sleep disturbance symptoms may be affected by non-pandemic factors not measured in the study. Factors such as academic stress, alcohol use, and the racial injustice brought to light during this time may be related to the observed increases in anxiety, depression, and sleep disturbances. Further studies should seek to better understand how impaired sleep and mental health interact during periods involving chronic stressors, like the global COVID-19 pandemic.

## Conclusions

Our study sheds light on the complexity of mental health issues among university students during the COVID-19 pandemic. The strengths of our study include the measurement of pandemic effects in the same individuals, pre- and during the COVID-19 pandemic and the use of both well-validated behavioral surveys and objective sleep measures. The results of our study suggest that the development of strategies for improving the mental health of young adults should target sleep behaviors as well as psychological and physiological stressors. Given that pandemic-associated factors have exacerbated many mental health challenges already prevalent in young adults, it is imperative that public health policies include initiatives to address accessibility to mental health services and education for vulnerable groups who are at increased levels of risk during the COVID-19 pandemic.

## Supporting information

S1 File (DOCX)

S2 File (CSV)
