# Supplementary material for: Intra-individual impact of the COVID-19 pandemic on mental health and sleep in young adults
Source: PLoS One. 2022 Oct 27;17(10):e0276165. doi: 10.1371/journal.pone.0276165 (PMC9612502; doi:10.1371/journal.pone.0276165)
Supplement: S1 File — (DOCX) [file pone.0276165.s001.docx]

**Supplementary File 1. Additional questions pertaining to sleep and mood pre- and during pandemic.**

*1. Relative to pre-pandemic:*

-I spend more/less/same time for sleep.

-I go to sleep earlier/later/same time.

-I feel my sleep was more/less/same in terms of health.

*2. During this pandemic*, I feel the following: (choose all that apply)

-hard to get to sleep

-stress and anxiety

-don’t have enough sleep

-don’t have constant sleep schedule

-have more vivid dreams/nightmares

-sleep disorder

-nothing
